# The effects of arts and crafts therapy on post-stroke executive dysfunction: a pilot randomized control test

**DOI:** 10.3389/fstro.2023.1242724

**Published:** 2023-08-16

**Authors:** Ruisheng Yun, Huanxia Zhou, Julie McLaughlin Gray, Jia Cheng, Zhongzhi Zhao

**Affiliations:** ^1^Department of Mental Health Rehabilitation Medical Center, Peking University Sixth Hospital/Institute of Mental Health, Beijing, China; ^2^Chan Division of Occupational Science and Occupational Therapy, University of Southern California, Los Angeles, CA, United States; ^3^Department of Rehabilitation Medical Center, Seventh People's Hospital of Shanghai University of Traditional Chinese Medicine, Shanghai, China

**Keywords:** arts and crafts therapy, stroke, executive function, rehabilitation, cognition

## Abstract

**Background:**

Executive function is an important determinant of independent living among stroke survivors. Patients with post-stroke executive dysfunction (PSED) have a lower engagement in therapy activities and reduced independent living abilities. One potential method for improving executive function and engagement is arts and crafts therapy (ACT). This study aimed to explore the effects of ACT on patients with PSED.

**Methods:**

The study was a pilot randomized controlled trial (RCT) with two groups: an experimental intervention group receiving ACT combined with individual rehabilitation therapy (IRT) and an active control group receiving IRT only. Fifty-seven patients with PSED participated. Outcome measures included the Trail Making Test (parts A and B), the Stroop test, the Tower of Hanoi (TOH), and the Lawton–Brody Instrumental Activities of Daily Living Scale. Two groups were compared at baseline and 4 weeks after the completion of the training.

**Results:**

There were significant differences in the Trail Making Test part A (TMT-A; time; *p* < 0.01), the TMT part B (TMT-B; *p* < 0.05), the TMT-B (errors; *p* < 0.01), and the Stroop test (time; *p* < 0.01) between the experimental and the control group after 4 weeks. There were no significant differences in TMT-A (errors), Stroop test (errors), TOH, and Instrumental Activities of Daily Living Scale.

**Conclusion:**

The therapeutic use of arts and crafts could be an effective intervention to improve executive function and self-efficacy for stroke survivors.

**Clinical trial registration:**

http://www.chictr.org.cn; Identifier: ChiCTR2200063547.

## Highlights

- Post-stroke executive dysfunction (PSED) should not be ignored during occupational therapy.
- Since the time and place of arts and crafts therapy are optional and not clinically limited, it should be put into practice to improve poststroke executive function.

## Introduction

Stroke remains one of the highest leading causes of death and long-term disability (Virani et al., 2020). There is increasing evidence of poststroke cognitive decline among survivors, with a prevalence of 38%, and this cognitive decline was one of the most significant factors contributing to long-term disability and dependence (Huang et al., 2022). Cognitive functions, especially executive function, play a crucial role in predicting poststroke long-term rehabilitation outcomes (Shea-Shumsky et al., 2019). *Executive function* is an umbrella term for a variety of higher cognitive functions, of which working memory, flexibility, and inhibition are the core elements (Povroznik et al., 2018). Successful independent living requires the ability to independently perform instrumental activities of daily living (IADLs), such as cooking, shopping, financial management, and medication management (Ghaffari et al., 2021). Unfortunately, poststroke executive dysfunction (PSED) affects more than 75% of survivors, making it challenging for them to perform these complex living tasks and to adversely impact their quality of life (Povroznik et al., 2018). In addition, patients with PSED demonstrate lower rates of functional recovery due to their misunderstanding of the treatment instructions and lower attendance in therapy activities due to attention deficits and decreased self-efficacy (Park et al., 2015; Shao et al., 2020). Consequently, individuals with PESD are at a higher risk of recurrent stroke or even death (Kwan et al., 2021), potentially leading to prolonged hospital stays and further ongoing expenses.

While various cognitive rehabilitation approaches have been investigated with mixed results (Chung et al., 2013), it remains unclear if these interventions have clinical effectiveness in improving executive function, IADL performance, and community participation (Gibson et al., 2022). Arts and crafts therapy (ACT) is a cost-effective and highly efficient alternative intervention that has received limited research attention and could potentially improve cognitive functions and motivate patients' participation. ACT provides intellectual stimulation and has been considered advantageous for neuronal processes underlying cognitive impairment (Camic et al., 2016). Furthermore, ACT involves using skillful craft activities that are motivating and engaging to enrich the lives of individuals, families, and communities (Tubbs and Droke, 2016). Notably, the art-making processes require the demonstration of executive abilities such as planning, decision-making, shifting, emotional regulation, and problem solving to address the challenges met in the process and complete the final crafts.

While some studies have reported improvements in general cognitive function and self-reported executive function with ACT, the cognitive performance of executive function has been less explored (Abbing et al., 2019). Similarly, a few qualitative studies have highlighted the benefits of arts and crafts in improving cognitive function, self-confidence, quality of life, and wellbeing among stroke survivors (Reynolds et al., 2008; Beesley et al., 2011). However, these studies have low certainty, and it remains unclear whether the results show clinical significance for use with patients with PSED.

This quantitative study aims to address this evidence gap by exploring the effects of stroke survivors' engagement in ACT on their cognitive performance aspects of executive function and participation experience.

## Methods

### Subjects and inclusion criteria

A small sample of 50 subjects was created based on an alpha set at 0.05, a power of 0.8, and previous similar pilot trials in ACT (Hattori et al., 2011).

Subjects were recruited from the inpatient and outpatient rehabilitation center of the Seventh People's Hospital of Shanghai University of Traditional Chinese Medicine through posters on social media and hospital chalkboards. All subjects provided informed consent for this study.

The inclusion criteria included (1) stroke diagnosed by computed tomography or magnetic resonance imaging, (2) primary stroke and course of disease (1–12 months), (3) cognitive dysfunction with Montreal Cognitive Assessment (MoCA) Beijing version score of <26 points and executive function subtests in MoCA of <5 points, and (4) self-reported or caregiver-reported difficulties with IADLs.

The exclusion criteria were as follows: (1) accompanied by severe mental illness; (2) severe cognitive dysfunction with the MoCA scores of < 10; (3) receiving other cognitive interventions including medication or therapy; (4) accompanied by moderate or severe difficulties of visual, hearing, and verbal expression or handwriting; (5) the patient's medical condition was unstable, or they were unable to maintain sustained attention for at least 15 min.

### Ethical approval

This study obtained ethical approval from the ethics committee of the Seventh People's Hospital of Shanghai University of Traditional Chinese Medicine (reference number 2022-7th-HIRB-015) on 7 July 2022. This research was registered at Chictr.org (Chinese Clinical Trial Registry Unique Identifier: ChiCTR2200063547) on 10 September 2022.

### Study design and intervention

Fifty-seven subjects who met the inclusion criteria participated in this study. All subjects were randomly assigned based on computerized random numbers in a 1:1 ratio to two groups: an experimental intervention group receiving ACT and individual rehabilitation therapy (IRT) and an active control group receiving IRT only. The therapists responsible for IRT were blinded, and all subjects were assessed by a blinded occupational therapist before and after the 4-week intervention.

Both the experimental intervention group and the control group received twenty 30-min sessions of IRT over 4 weeks. The IRT sessions included strengthening exercises, gross and fine motor training, and balancing exercises for the recovery of hemiplegia limb function. These sessions were provided by licensed and trained physical therapists.

**Figure 1:**
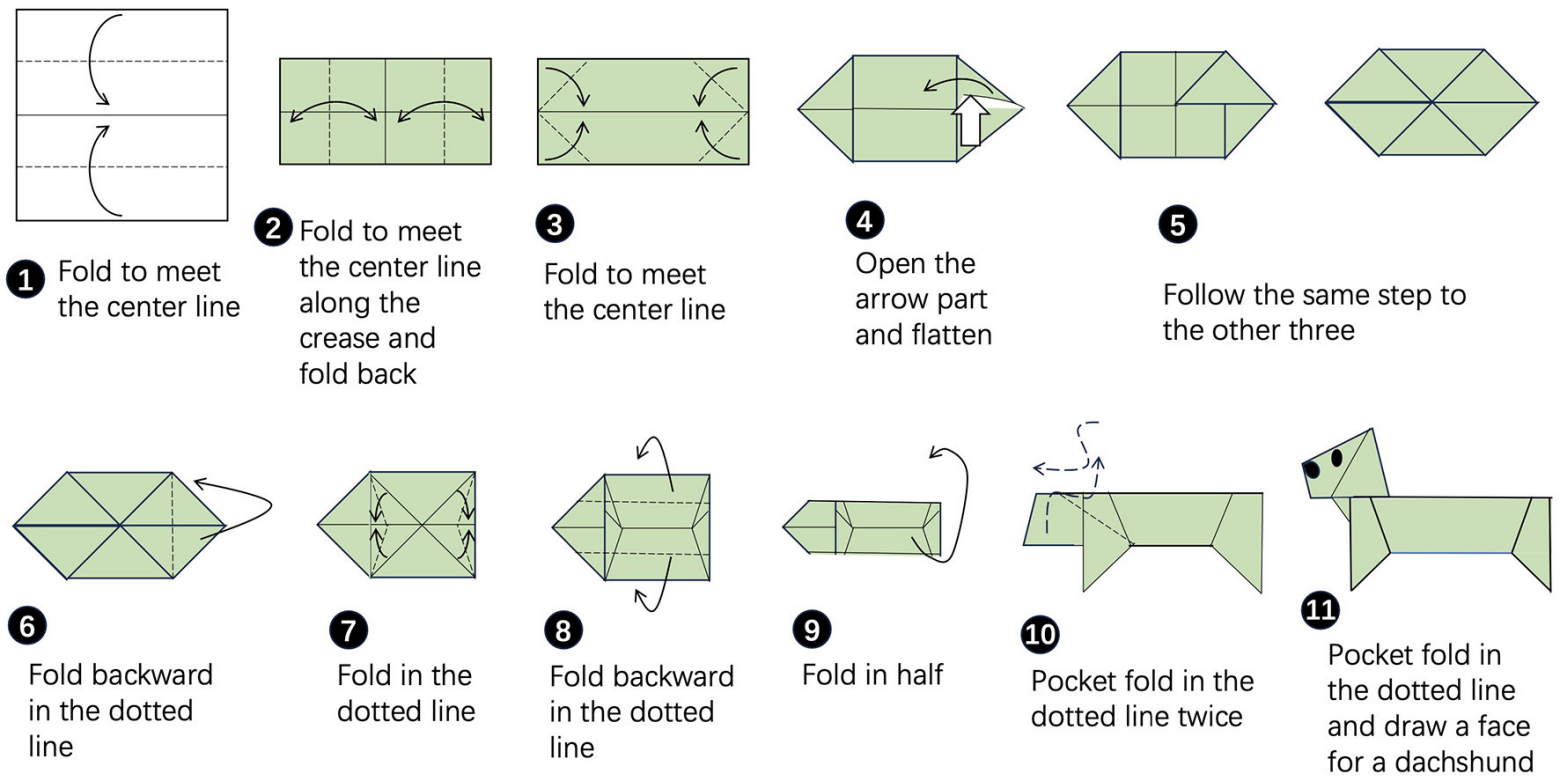
In the art of origami, subjects would create a dachshund from a sheet of paper using multistep folds and creases.

In the active control group, all subjects received an additional twenty 30-min sessions of IRT that consisted of a range of occupational therapy, including flexibility training, coordination training, repetitive task training, and activities of daily living (ADL) training. In the experimental group, subjects received twenty 30-min sessions of ACT. The ACT sessions were conducted in a group format, with 10–11 patients, in a quiet and relaxing rehabilitation setting. Each session followed a consistent structure, beginning with a 5-min welcome, followed by a 20-min art-making activity facilitated by two trained occupational therapists. The art-making activities were multistep activities (see Figure 1 as an example), and all subjects engaged in one activity at each session based on an activity menu throughout the whole week. The menu included origami on Monday, painting on Tuesday, paper cutting on Wednesday, coloring on Thursday, and calligraphy on Friday. The occupational therapist was in charge of laying out the supplies before the session started. During the session, the occupational therapist played a coaching role by encouraging subjects to use their affected limbs and follow the step-by-step instructions to complete the crafts. The sessions ended with a 5-min closing circle, where all subjects could present their artwork to the other group members and engage in a debriefing to explore the cognitive and executive function domains utilized during the activity.

### Measurements

The MoCA Beijing version (Wang and Xie, 2006) was used to screen the general cognitive and executive dysfunction among patients with stroke. A total score on the MoCA of <26 and missing any scores on the executive subtests were considered as reflecting the existence of mild or even cognitive and executive dysfunction.

The Trail Making Test (TMT) was a classic test for working memory and flexibility (Shao et al., 2020). For the TMT part A (TMT-A), the subjects were required to connect 25 numbers (1–25) that were distributed randomly in increasing order as quickly and accurately as possible. For the TMT part B (TMT-B), the subjects were required to connect 13 numbers (1–13) and 13 letters (A–L) that were distributed randomly in an alternating, increasing order (i.e., 1–A−2–__–__–__). The time and errors were used to evaluate subjects' executive function, with less time and fewer errors being better.

The Stroop test (Stroop, 1935) was used to evaluate the individual's working memory and inhibition. The Chinese version of the Stroop test was found to be a reliable instrument and was used in this study (Wang et al., 2018). There were two subtests with two pages of 20 words in each. Page 1 was printed with “*blue, green, red*” in Chinese characters in an ink color that matched the word (e.g., the word “*blue*” was shown in blue). The subject was asked to read the words. Page 2 was printed with “*blue, green, red*” in Chinese characters in ink colors incongruent with the word (e.g., the word “*blue*” was shown in green). The subjects were asked to tell the ink colors of the words. The subjects were asked to read as quickly and accurately as possible and had only one chance to finish these tests. All subjects were given 2 min between the two subtests as a rest interval. Considering that page 2 requires higher ability to inhibit cognitive interference and executive function (Scarpina and Tagini, 2017), the reaction time and the number of errors on page 2 were used to assess subjects' performance, also with less time and fewer errors being better.

The Tower of Hanoi (TOH) was used to evaluate the individual's abilities in working memory, planning, and problem-solving (Goel and Grafman, 1995). The test began with five disks of different sizes (ABCDE, where A was the smallest and E was the largest) on three cylinders; disks B, C, and D were on the left cylinder, disk E was on the middle cylinder, and disks A was on the right cylinder. The subjects were required to move the five disks according to certain rules and finally place the disks on the middle cylinder in the order from the largest to the smallest (E–A). The rules were as follows: (1) only one disk could be moved at a time, (2) all disks should be placed on the cylinder, and (3) the larger disk should be placed below the smaller disk. The required time and steps taken to complete the test were used as measures, with less time and fewer steps being better.

The Lawton–Brody Instrumental Activities of Daily Living (IADL) Scale (Lawton and Brody, 1969) was an appropriate tool to assess individuals' functional status and independent living abilities. There are eight domains in the Lawton–Brody IADL scale: A. Ability to Use Telephone, B. Shopping, C. Food Preparation, D. Housekeeping, E. Laundry, F. Mode of Transportation, G. Responsibility for Own Medications, and H. Ability to Handle Finances. Subjects scored themselves on a scale from 0 (low function and dependent) to 3 or 4 (high function and independent) in these domains according to their highest level of functioning.

### Statistical analysis

The IBM SPSS 26.0 statistical program (IBM Corp, Armonk, NY, USA) was used for the statistical analysis. Continuous variables were checked for normality distribution by the Shapiro–Wilk normality test. When the variables conformed to a normal distribution, they were described as the means and standard deviations. Otherwise, they were described as medians with 25 and 75% quartiles. Categorical variables were described as frequencies and percentages. The experimental group and the active control group were compared at baseline. After the 4-week intervention, intragroup changes and intergroup differences were compared using one-way analysis of variance. Data that had not met normality distribution were compared using the Mann–Whitney *U*-test, and a *p*-value of < 0.05 was considered statistical significance.

## Results

### Demographic and clinical characteristics

**Figure 2:**
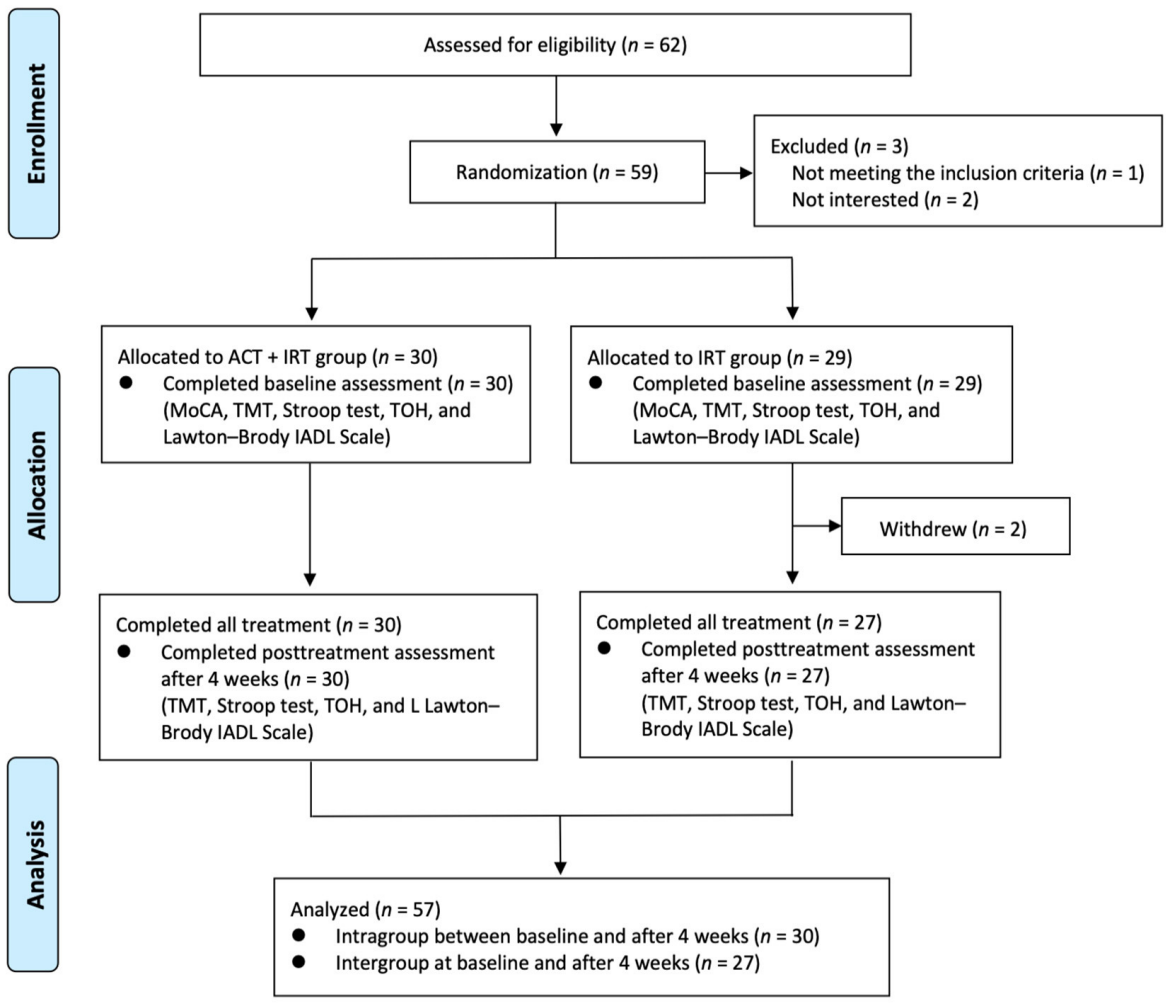
Flow chart showing the enrollment of patients and completion of the study. ACT, arts and crafts therapy; IRT, individual rehabilitation therapy; MoCA, Montreal Cognitive Assessment; TMT, Trail Making Test; TOH, Tower of Hanoi, IADL, Instrumental Activities of Daily Living.

A total of 57 patients, 33 males and 24 females, were enrolled in this study and finished all intervention sessions and measurements (Figure 2). Table 1 describes the demographic and clinical characteristics of all study subjects. No significant differences were found in baseline features between the two groups.

**Table 1:** Demographic and clinical characteristics of the experimental and control groups at baseline.

**Characteristics**	**Experimental group (*n* = 30)**	**Control group (*n* = 27)**	**Total (*N* = 57)**	***p*-value**
Age, mean (SD)	57.27 (15.61)	57.67 (14.79)	57.46 (15.01)	0.70
**Sex**, ***n*** **(%)**				
Male	17 (56.67)	16 (59.26)	33 (57.89)	0.843
Female	13 (43.33)	11 (40.74)	24 (42.11)	
**Stroke etiology**, ***n*** **(%)**				
Hemorrhage	15 (50.00)	17 (62.96)	32 (56.14)	0.325
Ischemia	15 (50.00)	10 (37.04)	25 (43.86)	
Course of disease, *n* (months)	7.17 (2.49)	7.19 (2.70)	7.17 (2.57)	0.979
**Affected hemisphere**, ***n*** **(%)**				
Left	11 (36.67)	10 (37.04)	21 (36.84)	0.941
Right	16 (53.33)	15 (55.55)	31 (54.39)	
Both	3 (10.00)	2 (7.41)	5 (8.77)	
MoCA, mean (*SD*)	21.06 (2.61)	21.07 (2.99)	21.07 (2.77)	0.574
Visuospatial/executive, mean (*SD*)	3.37 (1.16)	3.48 (1.22)	3.42 (1.18)	0.717
Naming, M (*p25, p75*)	3 (2, 3)	3 (2, 3)	3 (2, 3)	0.606
Attention, M (*p25, p75*)	3.5 (3,4)	4 (3, 5)	4 (3, 4)	0.522
Language, M (*p25, p75*)	2 (1.75, 2)	2 (1, 2)	2 (1, 2)	0.647
Conceptual thinking, M (*p25, p75*)	1 (1, 2)	1 (1, 2)	1 (1, 2)	0.915
Memory, mean (*SD*)	3.03 (0.85)	3.07 (0.96)	3.05 (0.89)	0.866
Orientation, M (*p25, p75*)	5 (5, 5.25)	5 (5, 5)	5 (5, 5)	0.722

MoCA, montreal cognitive assessment.

### Outcome measures

Table 2 describes the outcome measurement scores of all assessments. Before the 4-week intervention, the intergroup differences in the scores on the TMT-A, the TMT-B, the Stroop test, the TOH, and the Lawton–Brody IADL Scale were not significant (*p* > 0.05).

**Table 2:** Changes in outcome measures for the experimental and control group.

**Feature**	**IT**^**†**^ **ACT (mean**, ***SD*****)/(median, IQR)**			**IT (mean**, ***SD*****)/(median (IQR)**			***p*-value**
	**Pre-intervention**	**Postintervention**	Δ^†^	**Pre-intervention**	**Postintervention**	Δ^†^	
**TMT**							
TMT-a, times (s)	78 (61.75, 94.5)	62 (46,77.25)	−15.86 (8.61)^†*^	88 (72, 133)	87 (69, 108)	−7.8 (11.35)	0.006^**^
TMT-a, errors (*n*)	0 (0, 0)	0 (0, 0)	0 (0, 0)	0(0, 0)	0 (0, 0)	0 (0, 0)	0.928
TMT-b, times (s)	316.20 (115.83)	237.47 (106.65)	−69.05 (34.95)	336.63 (160.72)	339.93 (169.94)	3.30 (32.32)	0.008^**^
TMT-b, errors (*n*)	2 (0, 3)	1 (0, 3)	−0.48 (1.36)	3 (1, 4)	2 (1, 5)	0.04 (1.70)	0.032^*^
**Stroop Test**							
Times (s)	104 (91, 132.5)	92 (78, 105.75)	−23.05 (19.45)^†*^	127 (88, 147)	130 (99, 146)	7.04 (28.55)	0.004^*^
Errors (*n*)	3 (1, 8.25)	4 (1, 7)	−0.62 (0.86)	6 (1, 12)	5 (2, 12)	0.37 (2.08)	0.138
**TOH**							
Times (s)	393 (270.75, 606.25)	312 (113, 510.5)	−105 (−248, 42.5)^†*^	404 (255, 612)	425 (219, 612)	−9.22 (45.90)	0.089
Steps (*n*)	37 (21, 48.75)	31 (18.5, 52.25)	−0.76 (6.54)	41 (27, 80)	44 (27, 77)	0 (−2, 7)	0.083
Lawton-Brody IADL Scale	8.67 (3.43)	11.10 (4.22)	3.19 (1.99)	8.44 (4.55)	8.89 (4.82)	0.44 (1.53)	0.288

ACT, arts and crafts therapy; SD, standard deviation; IQR, interquartile range; TMT, Trail Making Test; s, seconds; TOH, Tower of Hanoi; IADL, Instrumental Activities of Daily Living.

† A comparison between postintervention of two groups.

* *p* < 0.05;

** *p* < 0.01.

Within the groups, the experimental group exceeded its pre-intervention scores on TMT-A time (*p* < 0.05), Stroop time (*p* < 0.05), and TOH time (*p* < 0.05). However, the active control group showed no significant changes compared with its pre-intervention outcome measures (*p* > 0.05).

**Figure 3:**
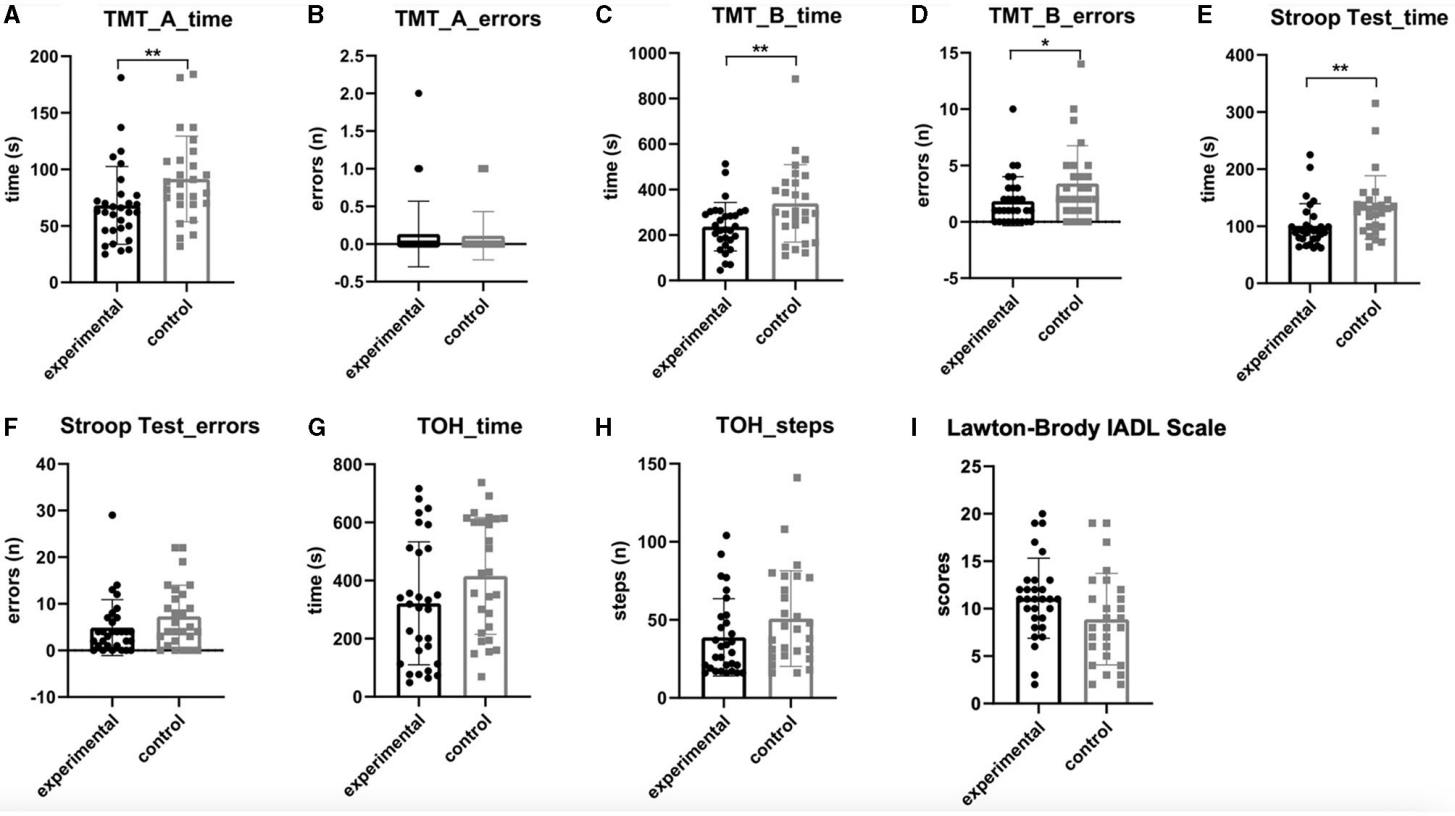
Comparison between the experimental group and the control group after the 4-week intervention. **(A)** TMT_A_time. **(B)** TMT_A_errors. **(C)** TMT_B_time. **(D)** TMT_B_errors. (E) Strrop test_time. **(F)** Stroop test_errors. **(G)** TOH_time. **(H)** TOH_steps. **(I)** Lawton-Brody IADL scale. TMT_A, Trail Making Test part A; TMT_B, Trail Making Test part B; TOH, Tower of Hanoi; IADL, Instrumental Activities of Daily Living. **p < 0.01; *p < 0.05.

After the 4-week intervention, the performance of the experimental group on TMT-A (time), TMT-B (time and errors), and the Stroop test (time) was significantly better than that of the active control group (*p* < 0.05). Statistical analysis showed no significance on TMT-A (errors), Stroop test (errors), TOH (time and steps), and Lawton–Brody IADL Scale (*p* > 0.05). See Figure 3 for more details.

## Discussion

The results of this study revealed that the IRT+ ACT group had significantly better scores on executive function assessments compared to the active control group. These findings provide quantitative evidence suggesting that ACT might enhance intervention plans for patients who have PSED. Additionally, implementing ACT is more flexible and cost-effective, with the supplies of the therapy costing under USD$ 10 per session. ACT can be implemented in both outpatient and inpatient settings, even with a short length of stay. Also, the group format of ACT provides equal face-to-face therapy time and peer support. Importantly, ACT can be administered in patients' homes, enabling them to practice independently in their familiar environments and experience comparable benefits.

The therapeutic use of arts was initially proposed by Adrian Hill in the 1940s and was first used in patients with tuberculosis and later in patients with stress disorders (Hogan, 2001; Stuckey and Nobel, 2010). Arts and crafts have since become prevalent in many clinical settings. They hold special therapeutic values as they afford purposeful and productive occupations that combine elements of recreation and therapy (Johnson, 1920). Additional benefits include providing pleasure, reducing negative emotions, enhancing self-esteem, and improving cognitive functions.

In Witkoski and Chaves's (2007) study, 11 participants diagnosed with Alzheimer's disease enrolled in painting or modeling ACT for 31 months. After the intervention, they had significant improvement in their cognitive functioning. Pike's (2013) and Yu et al.'s (2021) studies showed similar results; participants who had cognitive impairments performed significantly better in memory-related activities after art therapy. In addition, King et al. (2013) demonstrated that the therapeutic use of writing improved the self-confidence and cognitive function of 11 individuals diagnosed with mental health disorders in a pilot study. Nevertheless, there are only two studies that mention the benefits of ACT in improving the quality of life and wellbeing among stroke survivors (Reynolds et al., 2008; Kongkasuwan et al., 2016).

While this study indicates the results consistent with those of Beesley et al.'s (2011) qualitative study (Beesley et al., 2011), this study is the first quantitative study aimed at exploring both the clinical effectiveness and subjective satisfaction of ACT on patients with PSED. This study utilized a rigorous study methodology, with a control group, a relatively larger sample (*N* = 57), and a strict study design. In addition, pre-intervention and postintervention changes were evaluated comprehensively with multiple assessment tools based on the functioning and disability part of the International Classification of Functioning, Disability, and Health on multiple levels: body functions, body structure, activity, and participation. At the level of body functions and body structure, the TMT and the Stroop test were mainly used to evaluate individuals' brain structure and brain function; at the level of activity, the TOH and the Lawton–Brody IADL Scale were chosen to reflect individuals' performances on specific activities; and at the level of participation, the eight domains in Lawton–Brody IADL Scale were utilized to assess individuals' participation within their environment.

In the current study, the comparison of the two groups revealed significant differences on TMT-A (time), TMT-B, and Stroop test (time) between the experimental group and the active control group, whereas similar differences were not found in the outcomes of other assessments. This may be a result of the relatively low difficulty level of the TMT-A; almost all subjects in the two groups could finish the task without errors. However, the speed and efficacy of completion of the experimental were significantly greater than the active control group. We also compared within-group differences on the TMT-A and the TMT-B to determine differences in the performance of the same group of patients on the two tests and found significant differences in both time and errors (*p* < 0.01). Subjects in both groups performed worse on the TMT-B. The TMT-B is widely used to evaluate working memory and cognitive flexibility. In this study, the experimental group performed better in TMT-B and showed greater executive function than the active control group. Although errors on the Stroop test were not noted as significantly different between groups, the experimental group was more fluent. Thus, it might be considered that those in the experiment group had faster and better control of inhibition. The TOH is a challenging activity requiring high executive functioning. Although, between groups, the required time and steps of the TOH after the intervention were not statistically different, the experimental group gained significant progress on time to complete the TOH compared to their pre-intervention performance, and the active control group showed even worse results, suggesting that the experimental group performed better. Following the intervention, however, both groups did not demonstrate significant improvements in scores on Lawton–Brody IADL Scale. This was perhaps caused by the fact that progress in executive functions cannot be shown on this scale in a timely manner and has a certain lag. However, as expected, subjects who received ACT gained greater improvement compared to those in the active control group.

Stroke survivors, especially those with executive dysfunction, often show low self-confidence, low attendance, and low self-esteem resulting from living with reduced capabilities caused by physical and cognitive conditions (Robinson-Smith et al., 2000). ACT in this study involved creativity, skillful art-making process, and productive crafts, which had numerous benefits for patients with PSED. First, creative activities often include a multistep art-making process that can activate stroke survivors' brain lobes to organize and plan for complicated tasks (Johnson, 1920; Stuckey and Nobel, 2010). Second, ACT requires subjects to follow instructions to complete the tasks and track their own performance during the work, which are beneficial to working memory, inhibition, and task monitoring. Furthermore, during the art-making process, patients with PSED often pay more attention to their work, rather than to their symptoms or illness, which decreases negative emotions such as anxiety or depression (Kongkasuwan et al., 2016). In addition, ACT emphasizes the healing power of productive crafts (Tubbs and Droke, 2016). In this study, productive art materials were utilized as art mediums (i.e., coloring, painting, drawing, calligraphy, and paper cutting). Patients with PSED express likes or dislikes during the process and then produce their crafts to express themselves and connect with others (Papangelo et al., 2020). It is believed that viewing self-productive crafts activates patients' medial orbitofrontal cortex, which enables them to have rewarding and positive experiences (Ishizu and Zeki, 2011; Morelli et al., 2015).

Overall, ACT emphasizes holistic therapy and occupational engagement and therefore might be an effective and efficient rehabilitation for improving an individual's physical, cognitive, and psychological disabilities.

## Study limitations

Although this study complies with the principle of randomization, control, balance, and repetition as far as possible, the physical therapy was not completed by the same therapist due to clinical work stress. Fortunately, the interventions for the subjects were the same. Future studies should attempt to minimize any potential confounding elements by unifying treatment therapists between groups. Second, this study is also limited by a lack of follow-up evaluation; thus, the long-term effects of a combination of ACT and IRT on patients with PSED should be further explored. Finally, although this study utilized relatively comprehensive neuropsychological assessment tools, a lack of neuroimaging assessment was a limitation. Future studies with a larger sample size, longer intervention periods, and the integration of neuroimaging tools to further explore the mechanism of arts and crafts on patients with PSED are needed.

## Conclusion

This pilot study is the first quantitative study to explore the therapeutic effects of arts and crafts for patients with PSED. This study provides preliminary quantitative evidence that ACT could improve executive function among patients with PSED and should be considered for practice.

## Data availability statement

The raw data supporting the conclusions of this article will be made available by the authors, without undue reservation.

## Ethics statement

The studies involving humans were approved by the Ethics Committee of the Seventh People's Hospital of Shanghai University of Traditional Chinese Medicine. The studies were conducted in accordance with the local legislation and institutional requirements. The participants provided their written informed consent to participate in this study.

## Author contributions

RY was responsible for paper writing and data analyzing. RY and HZ were responsible for study design and data collection. JC and JG were responsible for paper writing and language polishing. ZZ was responsible for data collection, format checking and paper revisions. All authors contributed to the article and approved the submitted version.
